# Characterization of the function and clinical value of ERCC family genes in lung adenocarcinoma

**DOI:** 10.3389/fonc.2024.1476100

**Published:** 2024-11-08

**Authors:** Zhimin Lu, Guoxin Hou

**Affiliations:** ^1^ Department of Outpatient, Affiliated Hospital of Jiaxing University, The First Hospital of Jiaxing, Jiaxing, China; ^2^ Department of Oncology, Affiliated Hospital of Jiaxing University, The First Hospital of Jiaxing, Jiaxing, China

**Keywords:** ERCC genes, lung adenocarcinoma, bioinformatic, tumor infiltrating lymphocytes, ERCC8

## Abstract

**Introduction:**

ERCC genes, responsible for encoding enzymes involved in base excision repair, have been implicated in various cancers, contributing to chemoresistance. However, a comprehensive analysis of the prognostic and therapeutic significance of this gene family in lung adenocarcinoma (LUAD) is lacking.

**Methods:**

This study conducted a multidimensional assessment of ERCC family genes in LUAD using bioinformatic approaches, including mRNA expression level, gene methylation, and copy number variation (CNV), as well as their correlations with clinical outcome, gene set variations, and tumor-infiltrating lymphocytes (TILs). In addition, We evaluated the anti-tumor effects of ERCC8 in cell lines, demonstrating its clinical potential on an experimental level.

**Results:**

Overall, the expression of ERCC genes exhibited a negative correlation with good prognosis, with ERCC6L and ERCC8 demonstrating the most reliable predictive performance. Gene methylation level and CNV increases of ERCC genes generally displayed negative and positive associations with their expression levels, respectively. Additionally, GSVA analysis suggested that ERCC expression was positively correlated with cell cycle and apoptosis pathways but negatively correlated to the TSC/mTOR pathway. Furthermore, the expression of ERCC genes exhibited a complex relationship with TILs and the response to anti-tumor drugs. The results of in vitro cellular experiments show that inhibiting ERCC8 can alleviate the malignant phenotype of LUAD cells.

**Discussion:**

Our study revealed the multifaceted biological and clinical significance of ERCC family members in LUAD. These findings provide new insights into the function of ERCC family genes in LUAD and their potential clinical applications.

## Introduction

Lung cancer is a prevalent cancer worldwide, with the majority of patients presenting with metastasis and advanced stages at the time of diagnosis (1, 2). Lung cancer is broadly categorized into two clinical groups: small-cell lung cancer, and non-small cell lung cancer (NSCLC) (3). NSCLC constitutes over 80% of all lung cancer cases, with LUAD being the most frequently diagnosed subtype of NSCLC (4–6). Thus, LUAD is accountable for nearly half of all lung cancer-related deaths (7). Platinum-based chemotherapies are the primary treatments against LUAD (8). Nevertheless, resistance to these agents, either innate or acquired, is not uncommon, often attributed to increased DNA repair capacity in tumor cells (9).

Mutations in p53 have been identified as pivotal factors in chemotherapy resistance in lung cancer, influencing transactivation functions and the regulation of cell cycle and DNA repair mechanisms (10, 11). Additionally, the core components of representative DNA repair pathways, including DNA-dependent protein kinase catalytic subunits in the NHEJ pathway (12), TNKS1BP1 in homologous recombination (13), and XRCC1 in base-excision repair (14, 15) are implicated.

Excision repair cross-complementation (ERCC) enzymes are key proteins in the nucleotide excision repair (NER) pathway. Dysregulation of their expression has been demonstrated to participate in chemoresistance in various cancers. For example, ERCC1 and ERCC2 have been reported to be correlated with drug resistance in NSCLC (14, 15), ERCC2 controls the proliferation, migration and invasion of bladder cancer cells (16), elevated ERCC6 has been associated with drug resistance in colorectal cancer (17), and ERCC8 is involved in chemoresistance of esophageal cancer (18).

In addition to their correlation with decreased response to chemotherapies, the expression of ERCC family members, such as ERCC1, has also been positively correlated with cancer development (19, 20). Polymorphisms in ERCC genes are considered significant risk factors in various analyses (21–24), including those related to NSCLC (25–29), and the expression of ERCC6L is increased in tumor samples of various cancer types, including lung cancers (30, 31). However, the potential biological functions and clinical values of ERCC genes, including ERCC8, have not been systematically explored. Establishing a comprehensive understanding of the relationship between ERCC gene features and prognostic and oncologic characteristics can also facilitate the development of novel antitumor therapies and approaches against chemoresistance.

In this study, we conducted a comprehensive analysis of the internal correlation among the expression of ERCC genes, their methylation, copy number variations (CNV), and single nucleotide variations (SNV). Additionally, we explored how the expression profiles of ERCC genes related to cancer prognosis under various conditions, subtypes, immune cell infiltration, and the sensitivity of LUAD to different chemotherapeutic agents. Our findings revealed that high expression levels of ERCC genes, especially ERCC1, ERCC6L, and ERCC8 were significantly associated with poor prognosis. Gene set variation resulting from ERCC overexpression led to an increase in the activity of the cell cycle pathway, concurrent with the suppression of TSC/mTOR activity, and a reduction in the infiltration of cytotoxic cells. Moreover, increased expression of ERCC genes was negatively correlated with the sensitivity of LUAD to the majority of analyzed therapeutic agents. Experimental evidence was also provided to support the potential antitumor effect of ERCC8 knockdown in LUAD cells, highlighting its potential as a novel therapeutic target. Overall, we are the first to picture the landscape of ERCC family expression, variation, and potential biological function in the development of LUAD. This may offer insights into the diagnosis, characterization, and treatment of this fatal tumor.

**Figure 1:**
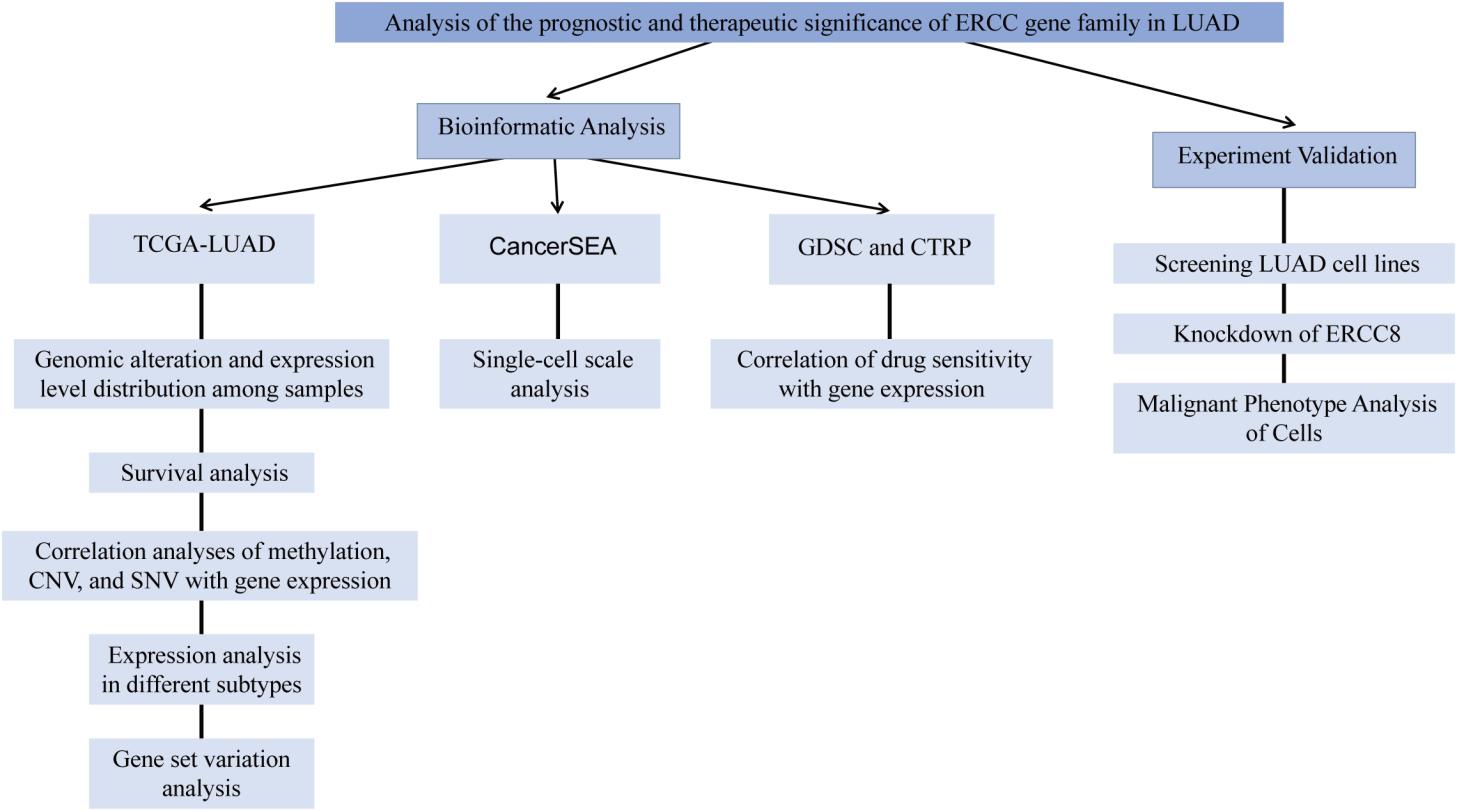
The flow chart of the study design and analysis.

The total study design is illustrated in **Figure 1**.

## Materials and methods

### TCGA expression analysis

ERCC family gene levels were obtained from TCGA (32) (https://portal.gdc.cancer.gov/), and the analysis comparing tumors and adjacent tissues was conducted using Student’s t-test.

### Survival analysis

The prognosis analysis of patients with LUAD was downloaded from the UCSC Xena project (http://xena.ucsc.edu), and the correlation between prognosis and ERCC gene expression levels was examined via univariate COX analysis using the R package “survival”.

### Gene set variation analysis

GSVA analysis, along with its correlation to enriched tumor-related pathways and tumor-infiltrating lymphocytes (TILs), was conducted using the GSCA online toolbox (33) (http://bioinfo.life.hust.edu.cn/GSCA/).

### Correlation of methylation, CNV, and SNV with gene expression

The correlation analyses were conducted using the ‘mutation’ module of the GSCA online database (33).

### Expression analysis in different subtypes

Expression difference analysis in different tumor subtypes was performed using the integrated tool TISIDB (34) (http://cis.hku.hk/TISIDB/).

### Correlation of drug sensitivity with gene expression

The correlation between drug sensitivity in GDSC and CTRP catalogs and ERCC family gene expression was performed using the ‘drug’ module of the GSCA online database (33).

### Genomic alteration and expression level distribution among samples

The cBio cancer genomics portal (35) was applied to display the distribution of CNV alterations and expression level changes among ERCC family genes in different LUAD samples.

### Single-cell scale analysis

Analyses of ERCC gene expression and t-SNE multi-dimension clustering of LUAD at the single-cell level were conducted using CancerSEA (36) (http://biocc.hrbmu.edu.cn/CancerSEA/home.jsp) using the EXP0066 dataset.

### Cell lines

All LUAD cell lines used in this study were purchased from Immocell Biotechnology (Xiamen, China), including BEAS-2B, NCI-H1395, NCI-H1975, HCC1833, NCI-H2009, and HCC827. All cells were cultured in DMEM (Gibco, Thermo Fisher Scientific) with 10% FBS (Gibco, Thermo Fisher Scientific) at 37°C with 5% CO_2_ and 95% air atmosphere with saturated humidity. All cells applied for experiments were examined to avoid Mycoplasma contamination using the Plasmo Test (InvivoGen).

### Vector construction and gene knockdown

For gene knockdown, three shRNAs against ERCC8 (shERCC8) and the negative control (shNC) were designed and lentivirus production was performed by Anti-hela Biological Technology (Xiamen, China) using the 4-plasmid system. Three shRNA-expressing lentivirus vectors were constructed for analysis. In the lentivirus infection process, HCC827 cells were plated and grown to 70% coverage, with media change as needed. The Virus was diluted in Opti-MEM and administrated to cells following the appropriate MOI. Virus-containing media were substituted into fresh media 24 h after virus addition. 60 hours after transfection, puromycin was added to the media at a final concentration of 3 μg/ml to eliminate the uninfected cells. Cells were harvested for further analysis 80 hours after transfection. The sequence of shRNAs and primers for shRNA vector construction are listed in **Table 1**.

**Table 1:** The sequence of primers for shRNA vector construction.

Name	Sequence (5'-3')	Target location (bp)
shERCC8-1-F	CCGGGCTGTGTATTTCAGTCAAATTCTCG AGAATTTGACTGAAATACACAGCTTTTT	1078-1098
shERCC8-1-R	AATTAAAAAGCTGTGTATTTCAGTCAAATTCTCGAGAATTTGACTGAAATACACAGC	1078-1098
shERCC8-2-F	CCGGGGGTCACAGACAAGAAATATTCTCGAGAATATTTCTTGTCTGTGACCCTTTTT	611-631
shERCC8-2-R	AATTAAAAAGGGTCACAGACAAGAAATATTCTCGAGAATATTTCTTGTCTGTGACCC	611-631
shERCC8-3-F	CCGGAGACAGAGATGTTGAAAGAATCTCGAGATTCTTTCAACATCTCTGTCTTTTTT	164-184
shERCC8-3-R	AATTAAAAAAGACAGAGATGTTGAAAGAATCTCGAGATTCTTTCAACATCTCTGTCT	164-184
shNC-F	CCGGTTCTCCGAACGTGTCACGTTTCTCGAGAAACGTGACACGTTCGGAGAATTTTT	–
shNC-R	AATTAAAAATTCTCCGAACGTGTCACGTTTCTCGAGAAACGTGACACGTTCGGAGAA	–

### RNA extraction and quantitative RT-PCR

RNA extraction from harvested cultured cells was performed using Trizol reagent (Invitrogen). Reverse transcription of RNA samples was performed using HiScript Reverse Transcriptase (Vazyme, China). qPCR was conducted on a LightCycler 96 instrument (Roche, USA) using ChamQ qPCR Mix (Vazyme, China). Three biological and technical replicates were performed in each group, and RNA levels were calculated using the 2^-△△Ct^ method. Primers for ERCC8 are listed in **Table 2**.

**Table 2:** Primers for ERCC8.

Name	Sequence (5'-3')	Target location (bp)
ERCC8-F	GCAGTTTCCTGGTCTCCACGTT	633-654
ERCC8-R	CAAACATCCTGATGCTCTTCTCAC	708-731

### Western blot

Protein samples were prepared from HCC827 cells using RIPA lysis buffer (Beyotime Biotechnology, China) according to the manufacturer’s protocol. Subsequently, protein samples were mixed with a loading buffer and denatured for 5 min at 95°C. Proteins were then separated by SDS-PAGE and transferred to PVDF membranes, which were blocked in a solution made from 4% nonfat powdered milk (Beyotime Biotechnology, China), and incubated with blocking solutions containing primary antibodies against ERCC8 (ab137033, Abcam, UK, 1:1000) or GAPDH (10494-1-AP, Proteintech, China, 1:5000) overnight at 4°C. After washing, the membranes were subsequently immersed in a blocking solution containing horse radish peroxidase (HRP)-conjugated goat anti-rabbit IgG (SA00001-2, Proteintech, China, 1:10,000) for 2h at room temperature. Finally, the membranes were rinsed with TBST and incubated with ECL reagent (Beyotime Biotechnology, China) for signal visualization. Protein levels were quantified after normalization to internal control using ImageJ (National Institutes of Health, USA). Three replications were performed for statistical analysis.

### MTT cell count assay

The MTT assay was performed using a commercial detection kit (Beyotime Biotechnology, China) following the instructions. Briefly, cells were seeded into 96-well plates at a density of 4,000 cells per well. 10 μL of MTT working solution (5 mg/mL) was added to each well, and the plates were incubated at 37°C for 6 hours. Subsequently, 100 μL of DMSO was pipetted into the plates to resolve crystallization, and the plates were kept for an additional 3 hours, followed by a 10-minute rotation at 37°C on an oscillator at 300 rpm/min. The absorbance value at 490 nm of each well was examined using a microplate reader. Biological triplicates were performed and analyzed for each condition for statistical analysis.

### Colony formation assay

Cells were seeded in 6-well plates at a density of approximately 700 cells per well and cultured for 12 days. Subsequently, the media was discarded, and the cells were fixed with 4% paraformaldehyde for 30 min and stained with 0.5% crystal violet for 15 min. The colonies were then washed, and photographed, and colony numbers were calculated using ImageJ. Three independent experiments were performed for each condition for quantification.

### Cell cycle and apoptosis analysis

For cell cycle analysis, 5×10^6^ HC827 cells were collected and treated with ethanol overnight at -20°C. Cells were then washed, resuspended in PBS, and treated with 20 μL RNase A for 30 min at 37°C. After the removal of RNase A, the cells were resuspended with PBS containing 5 μL 7-AAD solution (Thermo Fisher Scientific, USA) and kept in the dark at 4°C for 30 min. Finally, the cells were analyzed by flow cytometry using 488 nm excitation and 647 nm emission wavelength. Flow cytometry results were analyzed using Flowjo (BD Bioscience) to gate and calculate the proportion of cells in G0/G1, S, and G2/M phase.

For apoptosis detection using Annexin V-FITC/PI staining, Annexin V-FITC/PI apoptosis detection kit (Vazyme, China) was used following the manufacturer’s instructions. Briefly, 5 × 10^5^ HCC827 cells were harvested, washed in PBS, and resuspended with 100 μL of binding buffer from the kit. Then, 5 μL of Annexin-V FITC and 5 μL of PI solution were mixed and pipetted to the cells and incubated in darkness at room temperature for 10 min. After that, 400 μL of binding buffer was added, and cells were analyzed by flow cytometry using 488 nm as excitation wavelength. Annexin-V FITC or PI single staining was also performed for compensation adjustment. Flow cytometry results were analyzed using Flowjo (BD Bioscience) for gating and calculating the proportion of Annexin V-FITC^-^/PI^-^, Annexin V-FITC^+^/PI^-^, Annexin V-FITC^-^/PI^+^, and Annexin V-FITC^+^/PI^+^ cells. All flow cytometry experiments were performed in triplicate for statistical comparison.

### Transwell assay

Experiments were conducted using 24-well plates with 8 μm Transwell chambers (cat# 3422, Corning, USA). For invasion analysis, 100 μL of matrix glue was applied to each chamber, and the plates were kept at 37°C for 2 hours for preparation before cell transplantation. The cells were then digested, washed, and resuspended with serum-free media at 3×10^5^ cells per mL. After that, 200 μL of cell suspension was added to the top chamber and 700 μL of complete medium was added to the bottom chamber. After 48 hours of culture, the cells were fixed with methanol for 30 min at room temperature and then stained with 0.05% crystal violet (Solarbio, China) for 5 min. The plates were then observed and photographed under an inverted microscope (IX73, OLYMPUS, Japan). ImageJ was used for image processing and data quantification. For migration analysis, the experimental procedures were generally identical to the invasion experiment except that matrix glue was not applied. All the assays were repeated in triplicates.

### Statistical analysis

All comparisons between two sets of quantitative data were statistically analyzed using paired or unpaired Student’s t-tests. Experimental data were presented as mean ± SD. For bioinformatic analysis, correlations were calculated using the Pearson correlated coefficient, and group differences were analyzed by the Wilcoxon test.

*P* < 0.05 was considered statistically significant. *P* < 0.05, *P* < 0.01, *P* < 0.001, and *P* < 0.0001 were represented by *, **, ***, and ****, respectively.

## Results

### ERCC family members are generally overexpressed in LUAD

**Figure 2:**
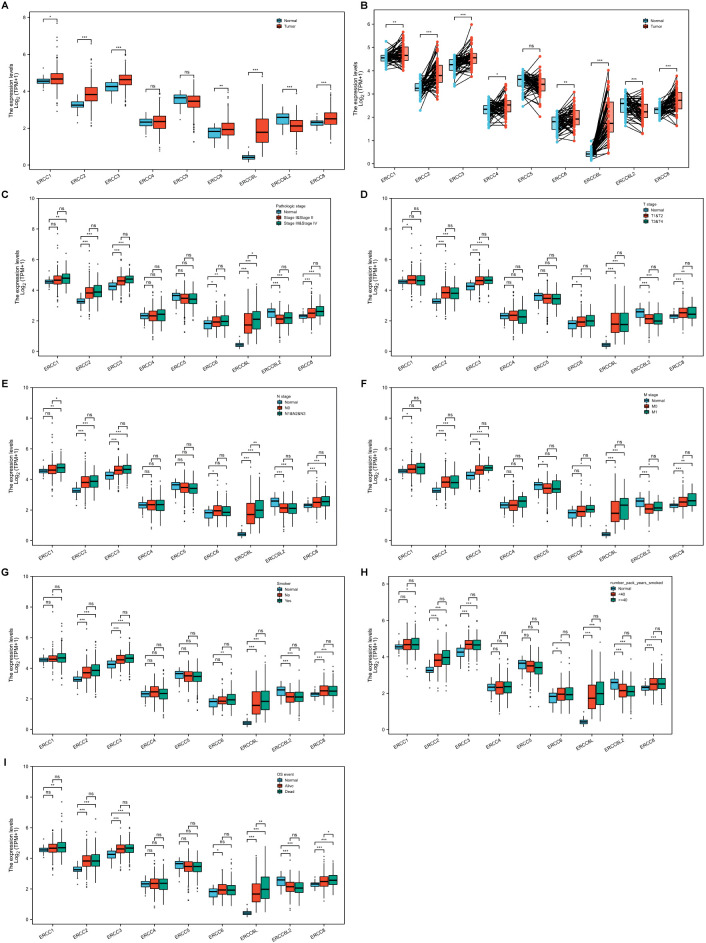
Expression levels of ERCC genes in LUAD. **(A, B)** Overall expression of ERCC genes in LUAD compared to adjacent controls in unpaired **(A)** or paired **(B)** analyses. **(C-I)** Comparison of ERCC gene expression levels in LUAD among different groups classified according to pathology stage **(C)**, T stage **(D)**, N stage **(E)**, M stage **(F)**, smoking **(G)**, smoke package year **(H)**, or OS **(I)**. *P<0.05, **P<0.01, and ***P<0.001.

We first assessed the expression levels of ERCC family genes, including ERCC1-6, ERCC6L, ERCC6L2, and ERCC8, using the TCGA-LUAD dataset. The findings indicated a general upregulation of ERCC genes when compared to controls, except for ERCC4, ERCC5, and ERCC6L2, which did not exhibit significant changes or showed a decrease (**Figures 2A, B**). To gain deeper insights, we further analyzed their expression, considering various factors such as pathology stage (**Figure 2C**), T stage (**Figure 2D**), N stage (**Figure 2E**), M stage (**Figure 2F**), and smoking behavior (**Figures 2G, H**). However, our results revealed only subtle or non-significant expression differences across this classification (**Figures 2C–I**). Notably, ERCC6L was the exception, showing upregulation in samples with metastasis compared to those without metastasis (**Figure 2E**). This finding is consistent with a previous report of ERCC6L’s involvement in epithelial-mesenchymal transition (37).

We then conducted an independent evaluation of the impact of patient gender, age, and race on ERCC gene expression in LUAD (**Supplementary Figures S1A–C**). Notably, we observed a decrease in ERCC6L expression in LUAD of aged patients, whereas ERCC6L2 and ERCC8 exhibited an increase in white LUAD patients. Furthermore, we explored the difference in ERCC gene expression in LUAD regarding patient survival, including overall survival (OS), disease-specific survival (DSS), and progress-free interval (PFI). Our analysis revealed that only ERCC6L displayed significantly higher expression in LUAD of dead compared to alive patients in all survival indexes, while ERCC1 and ERCC8 showed elevated expression in some cases (**Figure 2I**; **Supplementary Figures S1D, E**). In summary, the majority of ERCC genes exhibited upregulation in LUAD tissues compared to adjacent controls, with relatively minor influence from patient characteristics, survival index, or tumor progression stage. Notably, ERCC6L displayed the most significant differences.

Expression analysis using a single-cell dataset showed that all ERCC genes were significantly upregulated in LUAD (**Supplementary Figure S2A**), consistent with bulk expression analysis results of the TCGA dataset. To further determine the functional heterogeneity of the ERCC family in LUAD, we applied t-SNE clustering to the single-cell data and generated maps of gene expression clusters (**Supplementary Figure S2B**). These clusters suggested potential correlations with tumor cell heterogeneity and progression in LUAD.

### The prognosis value of ERCC family genes in LUAD

**Figure 3:**
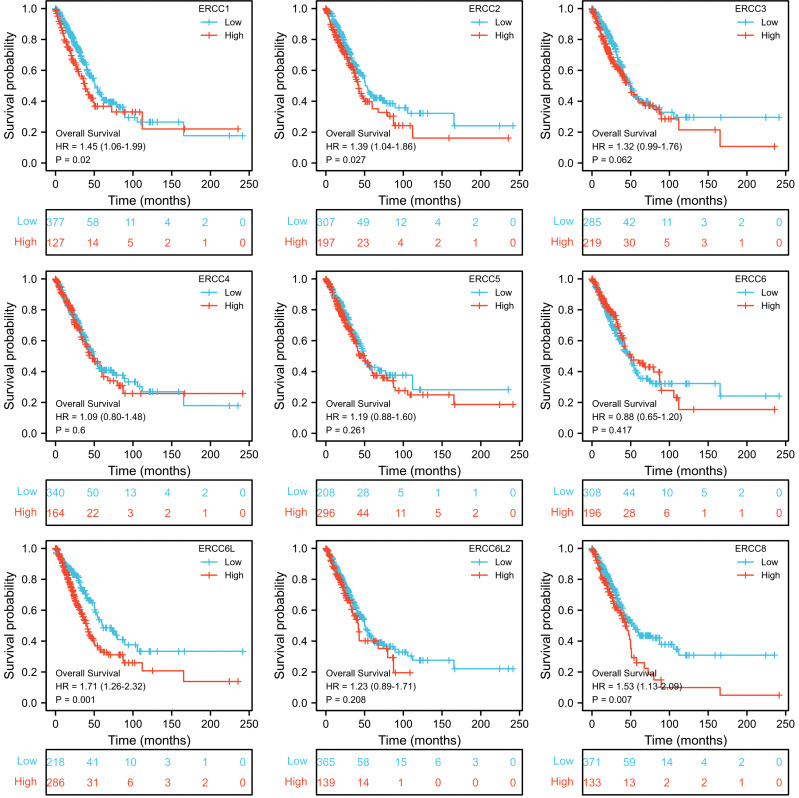
Correlation of ERCC family with LUAD patient’s prognosis using OS index. Kaplan–Meier curves indicating the OS probability of patients with high or low expression of ERCC genes were presented.

**Figure 4:**
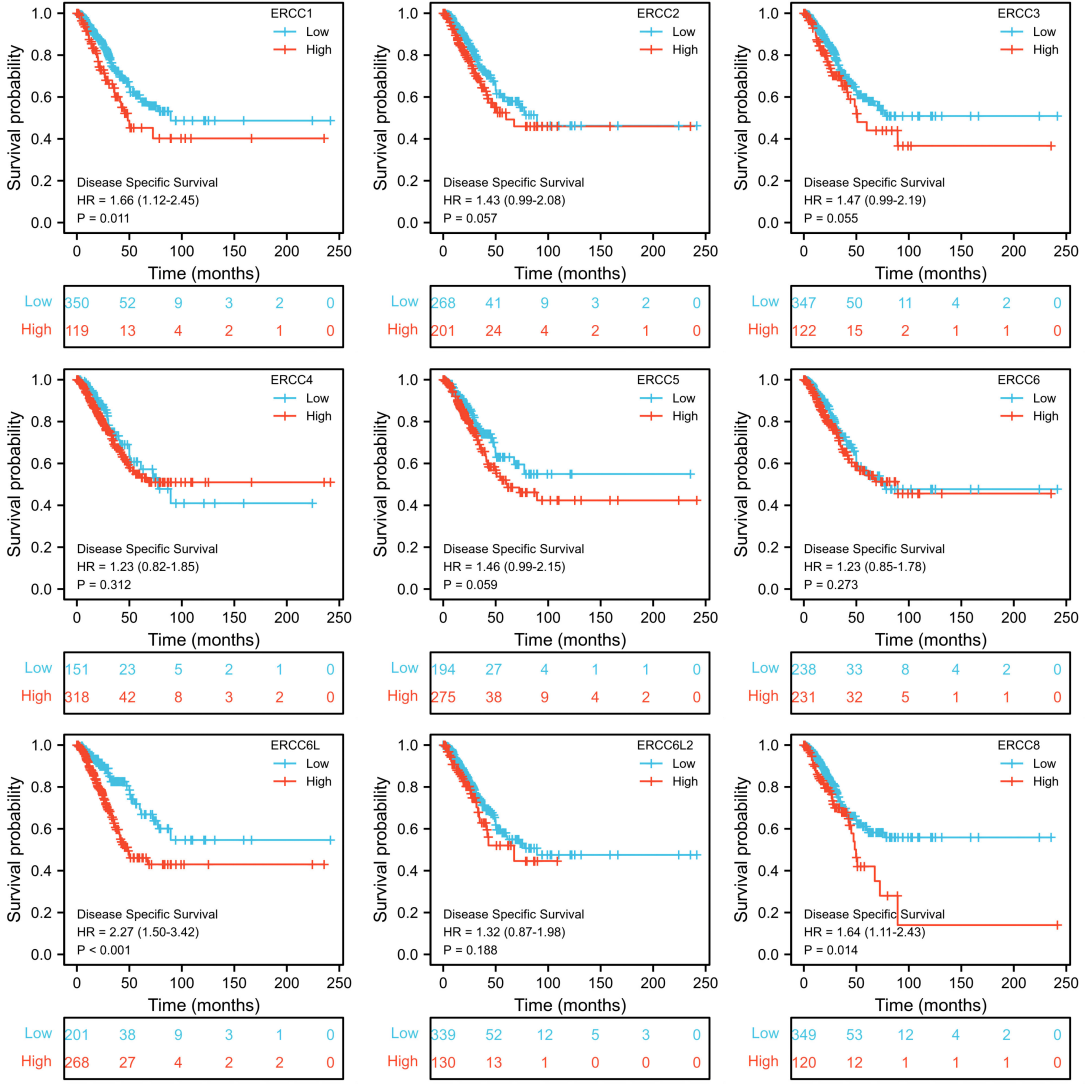
Correlation of ERCC family with LUAD patient’s prognosis using DSS index. Kaplan–Meier curves indicating the DSS probability of patients with high or low expression of ERCC genes were presented.

**Figure 5:**
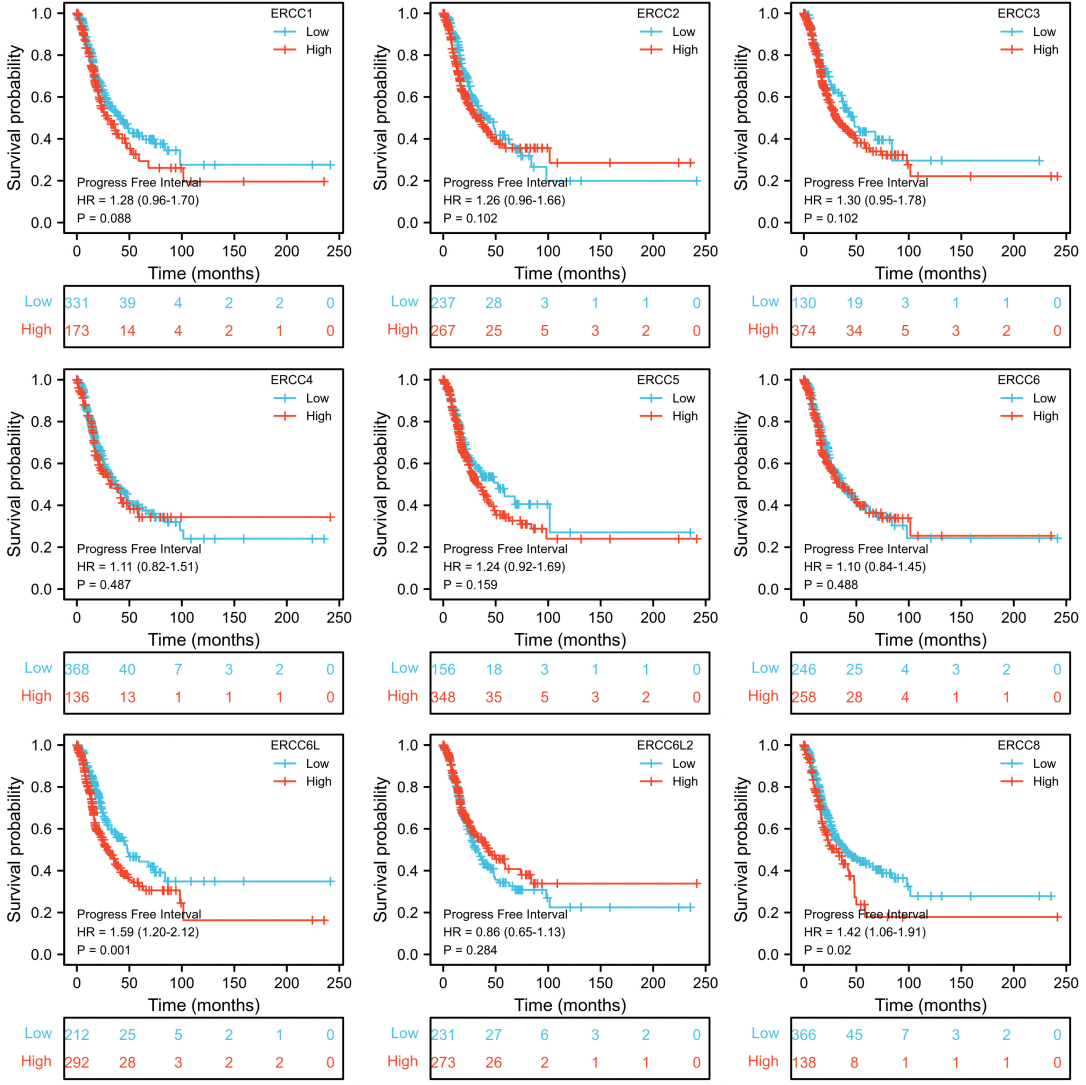
Correlation of ERCC family with LUAD patient’s prognosis using PFI index. Kaplan–Meier curves indicating the PFI probability of patients with high or low expression of ERCC genes were presented.

To assess the prognostic significance of ERCC gene expression, we employed Kaplan–Meier (KM) survival curves for patients with low or high expression levels of these genes. The results demonstrated that patients with higher expression of ERCC1, ERCC6L, and ERCC8 had a shorter OS or DSS compared with those with lower expression levels, while the differences in expression levels of the remaining genes showed less pronounced association (**Figures 3**, **4**). Furthermore, we investigated the impact of ERCC family genes on PFI and observed that only ERCC6L and ERCC8 maintained a significant correlation with expression levels, indicating their potent influence on cancer-related mortality (**Figure 5**). Taken together, among the ERCC family members, ERCC6L and ERCC8 emerged as the most robust predictors of LUAD prognosis.

### Genetic and epigenetic variations of the ERCC family in LUAD

**Figure 6:**
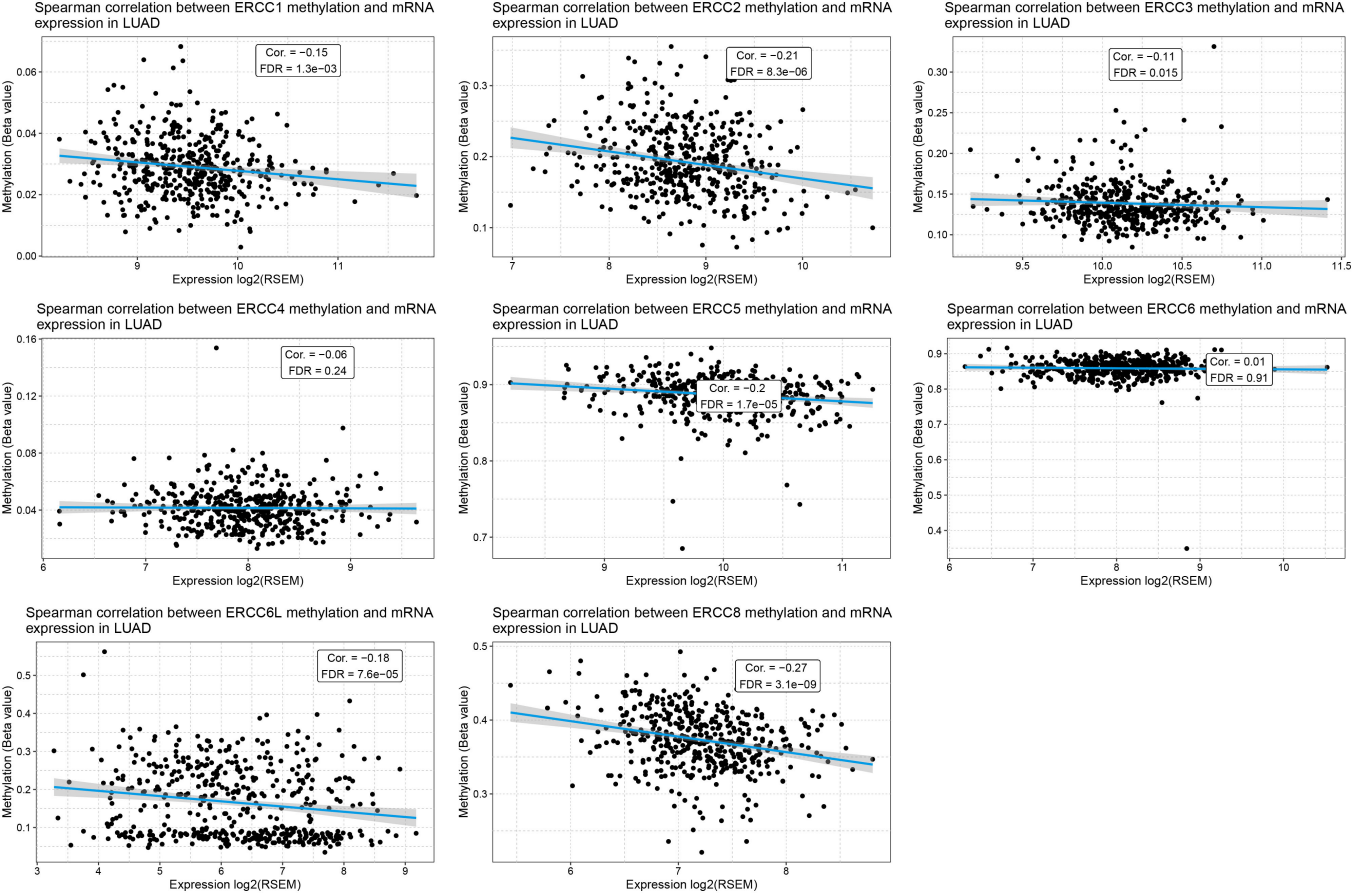
Methylation of ERCC genes negatively correlates with their expression in LUAD. Spearman correlation analysis between the methylation levels and mRNA expression levels for each ERCC gene in LUAD samples is displayed.

To unravel the underlying causes of ERCC gene regulation in LUAD, we investigated the correlation of gene expression levels and DNA methylation levels as well as CNV. As predicted, we found that DNA methylation levels of most of the ERCC genes exhibited a negative correlation with their gene expression levels in LUAD, including ERCC1, ERCC2, ERCC3, ERCC5, ERCC6L, and ERCC8 (**Figure 6**), with ERCC8 showing the lowest false discovery rate (FDR) (**Supplementary Figure S3A**). Notably, there is no available data on the DNA methylation of ERCC6L2, which warrants further investigation.

**Figure 7:**
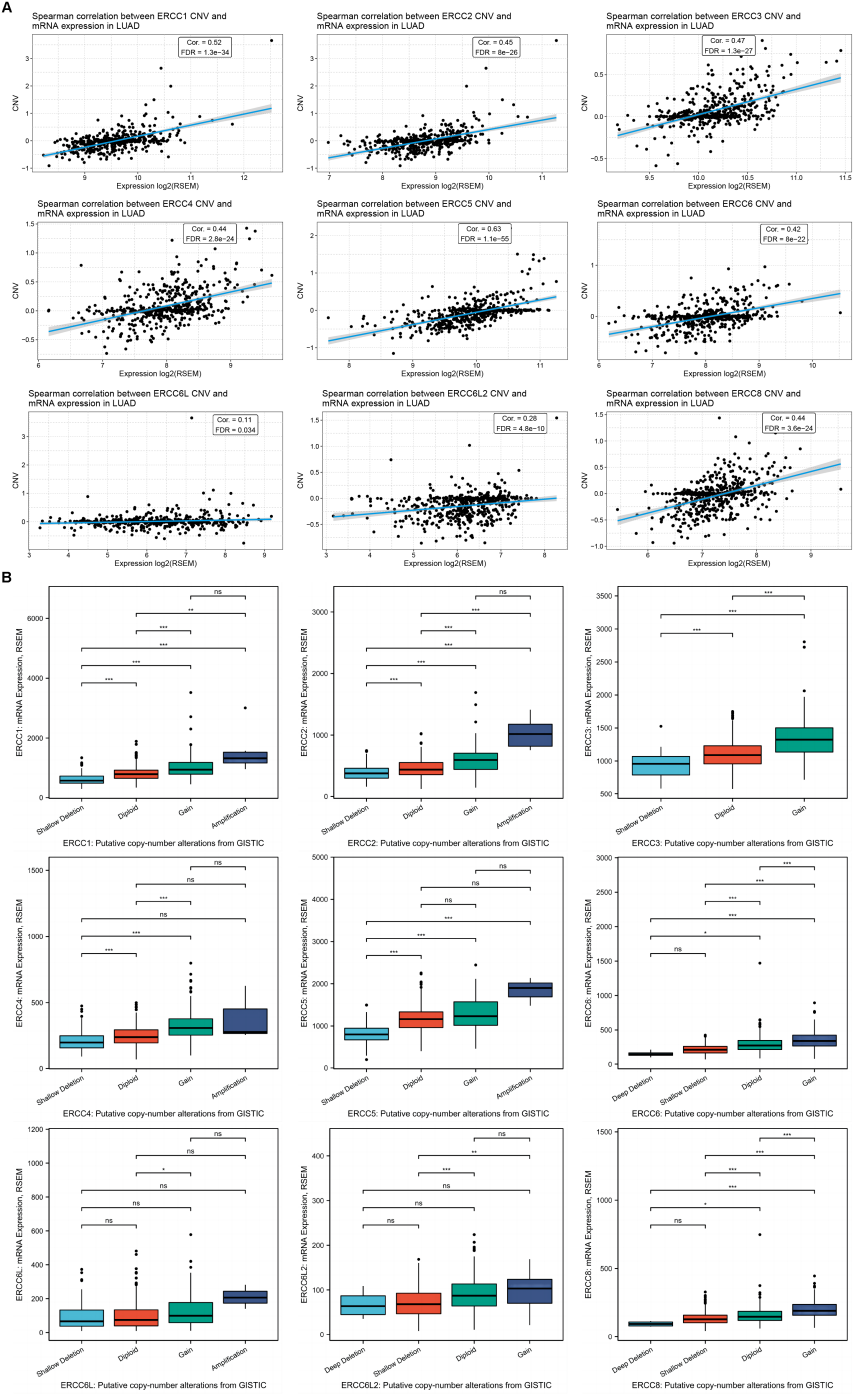
CNV of ERCC genes positively correlates with their expression in LUAD. **(A)** Spearman correlation analysis between the CNV and mRNA expression levels for each ERCC gene in LUAD samples is displayed. **(B)** RNA expression levels of the ERCC family under different gene CNV conditions of each gene. *P<0.05, **P<0.01, and ***P<0.001.

CNV, on the other hand, displayed a strong positive correlation with mRNA expression levels across all analyzed ERCC genes, except for ERCC3, due to the lack of relevant data in the LUAD dataset (**Figure 7**; **Supplementary Figure S2B**). The CNV distribution suggested that the majority of variations in the samples were heterozygous CNV, including amplifications and deletions, while the occurrence of homozygous CNV, especially homozygous deletion, was rare (**Supplementary Figures S2C, D**). The distribution of CNV, along with significant mRNA expression alterations, were shown in **Supplementary Figure S4**.

**Figure 8:**
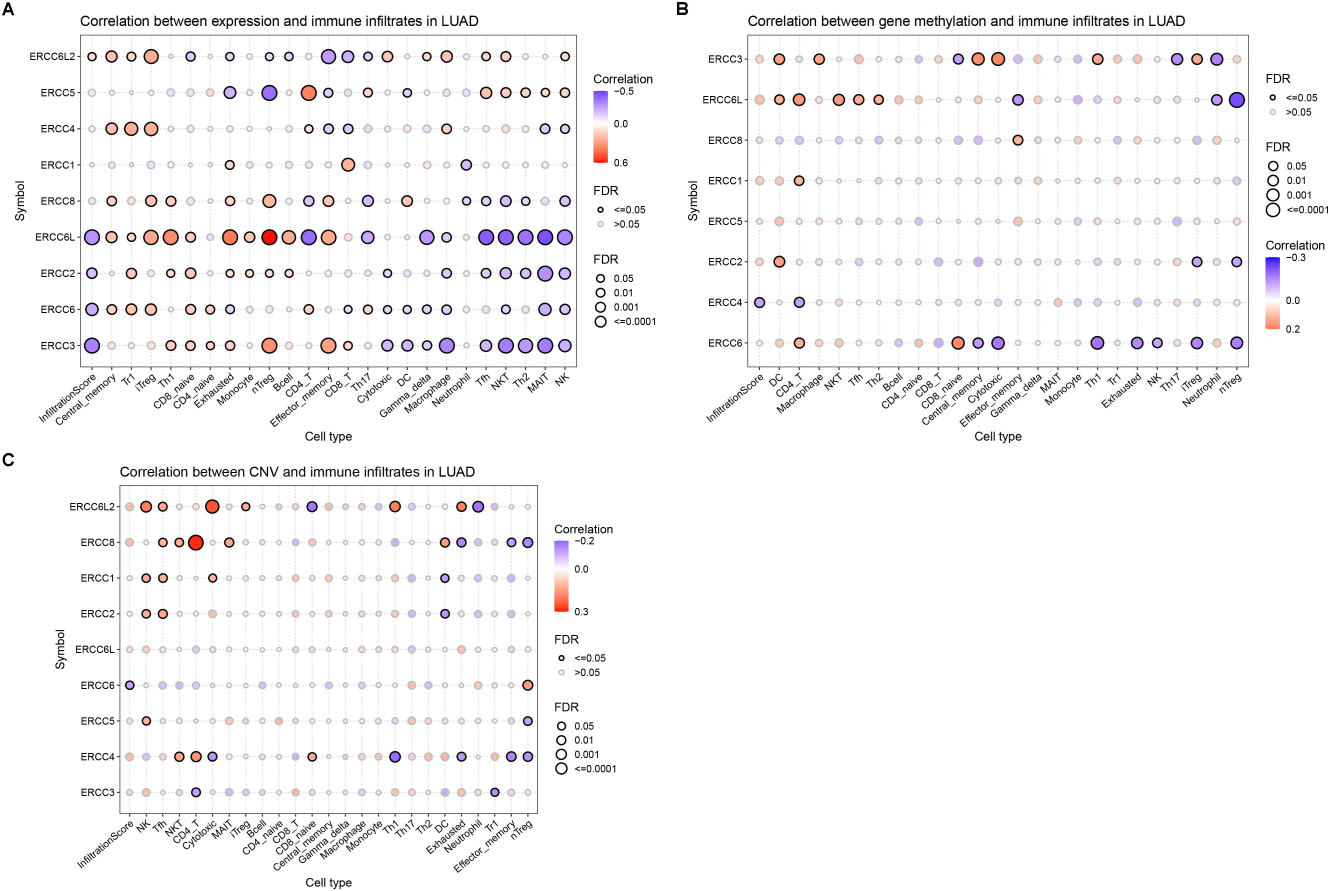
The correlation of ERCC family with LUAD TILs. **(A–C)**. Correlation analyses results of the infiltration of different leukocytes in LUAD with the mRNA expression level **(A)**, gene methylation level **(B)**, and CNV **(C)** of the genes in the ERCC family.

To delve deeper into the impact of CNV on ERCC gene expression and prognosis, we classified CNV cases into categories such as deletion, shallow deletion, diploid, gain, and amplification according to the copy number, and separately evaluated their influence. As expected, the groups with higher copy numbers displayed higher mRNA levels for most of the ERCC family members, except ERCC6L (**Figure 8A**).

### Correlation between ERCC family and immune cell infiltration and drug resistance in LUAD

To investigate whether ERCC family genes play a role in the distribution of TIL in LUAD, we applied the GSCA platform to examine the correlation between ERCC gene expression. We found that the overall infiltration of TIL was negatively correlated with the expression of ERCC6L, ERCC3, ERCC6, and ERCC2 (**Figure 8A**). Notably, the negatively associated TIL subtypes included cytotoxic cells, such as natural killer T (NKT) cells, natural killer (NK) cells, and gamma-delta T cells. Additionally, antitumor regulatory cells such as mucosal-associated invariant T (MAIT) cells, follicular helper T (TfH) cells, CD4 + T cells, and T helper 2 (TH2) cells also displayed negative correlations with the expression of these genes (**Figure 8A**). On the other hand, tumor-promoting cells, including naturally occurring Tregs (nTregs), adaptive/induced Tregs (iTregs), exhausted T cells, and T helper 1 (Th1) cells were positively correlated with the expression of these four genes (**Figure 8A**). Together, these results indicated the oncogenic function of the four ERCC genes.

Interestingly, although ERCC8 did not show an overall correlation with TILs, its correlation pattern with individual cell types was similar to those four genes with FDR below the threshold. This observation indicated the potential tumor-promoting function of ERCC8 expression by modulating leukocyte infiltration (**Figure 8A**). Conversely, ERCC4 and ERCC6L2 displayed distinct correlation patterns compared to ERCC6L and the other three genes, suggesting their tumor-suppressing function (**Figure 8A**). To further evaluate the effect of ERCC gene expression on LUAD immune subtypes, we performed a distribution analysis of their expression. This analysis revealed that expression levels of ERCC1, ERCC2, ERCC3, ERCC4, ERCC5, ERCC6, ERCC6L, ERCC6L2, and ERCC8 were significantly variated among different groups (**Supplementary Figure S5**). Specifically, ERCC6L and ERCC8 displayed increased expression in subtype C4, characterized as immunology quiet, suggesting that their expression may inhibit immune cell infiltration (**Supplementary Figure S5**). We also investigated the correlation between TILs and ERCC gene methylation and CNV, but only rare correlation events were observed (**Figures 8B, C**), indicating limited involvement of these characteristics in immune cell infiltration.

Additionally, we examined the association between ERCC family expression and sensitivity of chemotherapeutic agents sourced from GDSC and CTRP catalogs (**Supplementary Figures S6A, B**). We found that the majority of the genes displayed binary effects on the drugs analyzed, with both positive and negative correlations observed concerning the expression levels of ERCC1, ERCC3, ERCC4, ERCC6L, and ERCC8 (**Supplementary Figure S5A, B**).

### GSVA of the ERCC family

**Figure 9:**
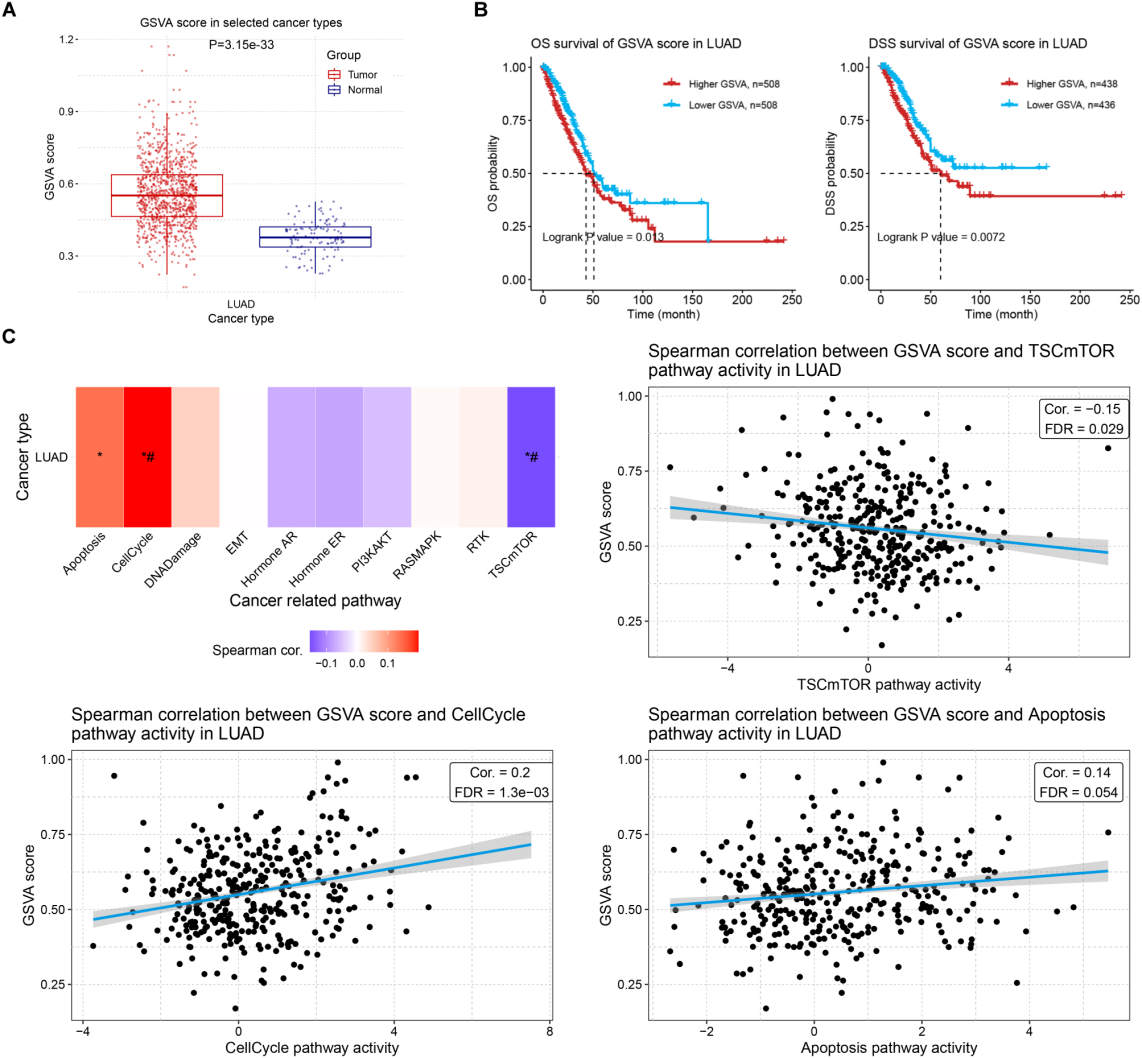
GSVA of ERCC family and its correlation with prognosis and tumor-related pathways. **(A)** GSVA scores of LUAD samples compared to controls. **(B)** Kaplan–Meier curves indicating the OS (left) or DSS (right) probability of patients with high or low GSVA scores of the ERCC family. **(C)** Spearman correlation analysis results between GSVA score of ERCC family and cancer-related pathways in LUAD. *: Cor > 0.1 or <-0.1; #: FDR < 0.05.

To evaluate the comprehensive impact of the ERCC family on LUAC, we performed GSVA analysis to examine their collective effect on tumor development. The GSVA score of the family was significantly higher in tumors compared to normal tissues (**Figure 9A**), which was consistent with the increased expression of most individual genes. Survival correlation analysis revealed a negative association between the GSVA score of the ERCC family and clinical outcomes (**Figure 9B**). To further investigate the effect of increased GSVA score on tumor-related pathway activity, we investigated their correlation and observed that the ERCC family was positively correlated with the cell cycle pathway but negatively correlated with the TSC/mTOR pathway (**Figure 9C**), which again demonstrated its oncologic function. Interestingly, the GSVA score of ERCC genes also positively correlated with apoptosis, albeit with a relatively high FDR (**Figure 9C**), suggesting that the ERCC family might simultaneously possess tumor-regulating functions.

**Figure 10:**
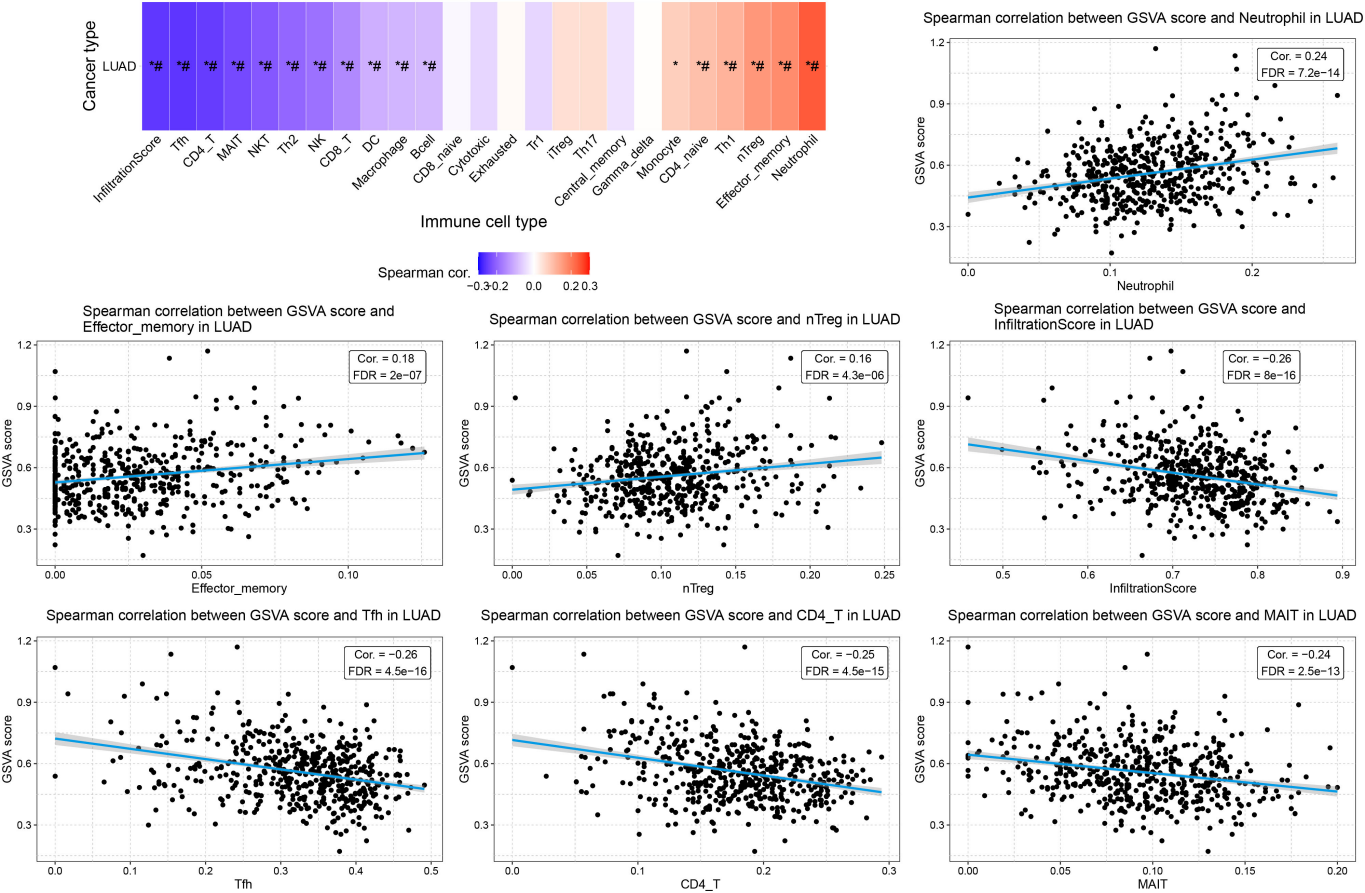
Association between GSVA score and TILs in LUAD. Spearman correlation figures displaying the top 3 positively correlated cell types, the top 3 negatively correlated cell types, and the total infiltration score. *: Cor > 0.1 or < -0.1; #: FDR < 0.05.

We also explore the correlation between the GSVA score of the ERCC family and TILs. Consistent with their correlations with TILs, ERCC genes collectively displayed a negative correlation with total infiltration, as well as infiltration of cytotoxic cells, antigen-presenting cells, and antitumor regulators (**Figure 10**, **Supplementary Figure S7**). Infiltration of tumor-promoting leukocytes, such as nTreg cells, showed a positive correlation with the GSVA score (**Figure 10**). Intriguingly, infiltration of neutrophils was also increased by ERCC family activation, indicating that ERCC genes may increase tumor inflammation of LUAD (**Figure 10**). Taken together, this GSVA analysis revealed the overall oncologic function of ERCC family genes. However, potential regulatory effects like apoptosis and inflammation were also observed, suggesting their complex role in LUAD.

### ERCC8 suppression alleviates tumor malignant phenotypes in LUAD cells

**Figure 11:**
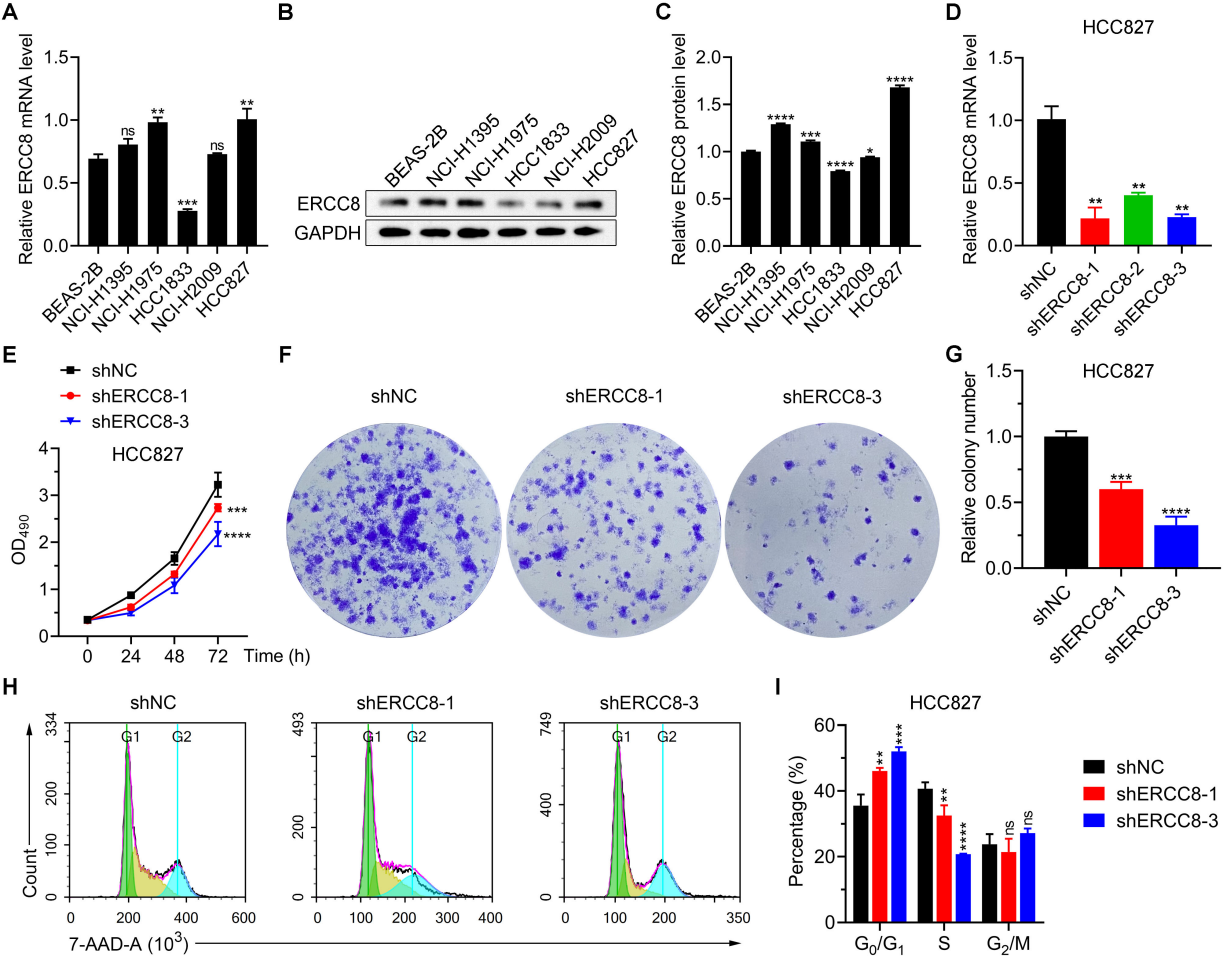
Inhibition of ERCC8 reduces LUAD cell viability and induces cell cycle arrest. **(A)** mRNA expression analysis of ERCC8 expression in BEAS-2B cells as well as five LUAD cell lines using RT-qPCR. B and **(C)** Representative image **(B)** and quantification **(C)** of ERCC8 protein expression in BEAS-2B cells as well as five LUAD cell lines. **(D)** RT-qPCR analysis of ERCC8 expression after different shRNA transfection in HCC827 cells. **(E)** MTT assay results showing HCC827 cell viability and proliferation after ERCC8 knockdown. **(F, G)** Representative images **(F)** and quantitative results **(G)** of clonal formation assay using HCC827 cells with or without ERCC8 suppression. **(H, I)** Representative fluorescence distribution **(H)** and cell phase quantification **(I)** of 7-AAD cell cycle staining in HCC827 cells after ERCC8 inhibition. *P<0.05, **P<0.01, ***P<0.001, and ****P<0.0001.

**Figure 12:**
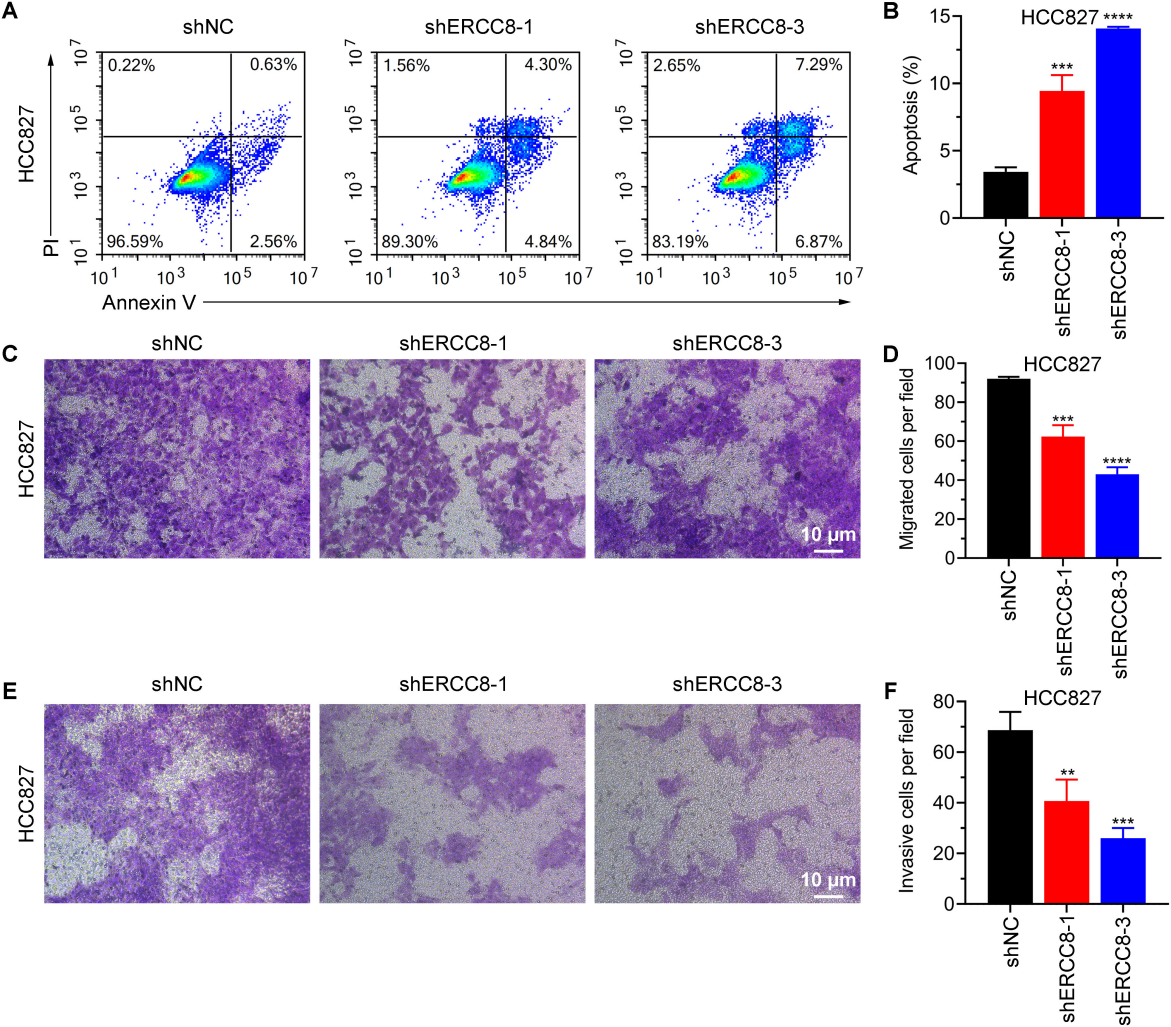
ERCC8 suppression promotes apoptosis and alleviates the migration and invasion capacity of LUAD cells. **(A, B)** Representative images of flow cytometry gating **(A)** and quantification of apoptosis **(B)** using Annexin V/PI staining of HCC827 cells after shRNA administration. **(C–F)**. Transwell migration **(C, D)** and invasion **(E, F)** results of HCC827 cells with or without ERCC8 knockdown. Both representative images **(C, E)** and quantitative analysis results **(D, F)** were displayed. **P<0.01, and ***P<0.001.

From previous bioinformatic analyses, we identified ERCC1, ERCC6L, and ERCC8 as having the strongest oncogenic effects in LUAD. Since experimental evidence regarding the functions of ERCC1 and ERCC6L in LUAD has already been reported (37, 38), we performed experiments to investigate the function of ERCC8 in LUAD cell lines. We first compared the expression levels of ERCC8 in five LUAD cell lines with those in the non-tumorigenic BEAS-2B cells. Among the five cell lines, HCC827 displayed the highest ERCC8 expression, both at mRNA and protein levels (**Figures 11A–C**). While ERCC8 expression was up-regulated in NCI-H1395, NCI-H1975, and HCC827 cells, it showed no significant change in NCI-H2009 cells and was reduced in HCC1833 cells. These findings collectively indicated that ERCC8 is highly expressed in most LUAD cases (**Figures 11A–C**). Due to high ERCC8 expression in HCC827, we focused on HCC827 for further experiments and designed shRNAs to effectively suppress its suppression (**Figure 11D**). Utilizing cell count and colony formation assays, we observed that ERCC8 knockdown significantly reduced cell proliferation capacity (**Figures 11E–G**). Moreover, ERCC8 knockdown resulted in an increased number of cells arrested in the G0/G1 phase and a decreased cell proportion in the S phase (**Figures 11H, I**), as well as enhanced apoptosis in HCC827 cells (**Figures 12A, B**). In addition, ERCC8 suppression impaired the migration and invasion capacity of LUAD cells, as indicated by the results of transwell assays (**Figures 12C–F**). Collectively, these results not only demonstrated the oncogenic function of ERCC8 in LUAD at the experimental level but also suggested its potential as a therapeutic target for antitumor therapy.

## Discussion

In this study, we systematically analyzed the clinical significance of ERCC family members, both individually and systematically, in LUAD. We observed that ERCC1, ERCC6L, and ERCC8 expression significantly increased in LUAD and were most strongly correlated with poor clinical outcomes. Moreover, we delved into the interplay between the expression of ERCC genes, their methylation patterns, and CNV, shedding light on their collective impact on TILs. Our investigations unveiled that the expression of ERCC genes showed a general tumor-promoting effect on cell infiltration. Furthermore, we performed GSVA analysis of the ERCC family, uncovering their correlations with crucial pathways, including cell cycle, apoptosis, and TSC/mTOR pathways, as well as clinical outcome and TILs, demonstrating a whole picture oncogenic function of the ERCC family in LUAD. Importantly, we also provided experimental evidence supporting the oncogenic function of ERCC8 through its inhibition.

While SNVs of ERCC genes, especially ERCC1 and ERCC2, have been widely studied for their involvement in the drug resistance of LUAD (14, 15), factors related to mutations or modifications beyond SNVs remain poorly explored. Our integrative analysis examined the relationship between methylation or CNV and mRNA expression of ERCC genes, as well as their impact on immune cell infiltration. This comprehensive approach suggested that variations other than SNPs in the ERCC family may also contribute to the oncogenic effects and chemotherapy resistance in LUAD. Furthermore, we expanded the investigation of ERCC gene expression and drug resistance to all ERCC genes, implying that the ERCC family might collectively contribute to drug resistance.

GSVA offers comprehensive information of expression information within a given gene set (39), making it highly suitable for analyzing the function of gene families in tumorigenesis. In our study, we performed GSVA and correlated it with tumor-related molecular pathways as well as TILs, providing insight into the overall impact of the ERCC gene in LUAD. The promotion of the cell cycle pathway and inhibition of TSC tumor suppressor by ERCC GSVA indicated the potential biological function of this gene family beyond base excision repair or regulation of the expression and function of these genes like TSC1 or TSC2. Surprisingly, an increase in ERCC GSVA scores displayed a positive tendency toward the activation of the cell apoptosis pathway, which is contradictory to experimental evidence that inhibition of ERCC1, ERCC6L, or ERCC8 promotes apoptosis of tumor cells (19, 37). Thus, a more detailed investigation into the role of the ERCC gene in apoptosis is needed.

The tumor environment and TILs dynamically regulate the progression of the tumorigenesis process (40, 41). To gain a comprehensive understanding of the impact of ERCC genes on TILs, we investigated the correlation of mRNA expression, CNV, methylation, and GSVA with infiltration of different immune cell types. A significant correlation was observed in the analysis of mRNA expression level and GSVA, both of which displayed favorable infiltration of oncogenic cells like Treg and Th1, as well as impaired infiltration of cytotoxic cells and some antigen-presenting cells. Further validation of these correlations is required especially on the clinical experiment level to bridge the correlation of genetic variations with tumor immune subtypes. Moreover, the underlying mechanism of how the ERCC family affects leukocyte infiltration requires further characterization, which may contribute to the development of novel genetic therapies against LUAD and other types of tumors.

Besides the comprehensive data analyses of the ERCC family, we also performed experimental investigations into the less-reported oncogenic function of ERCC8 in LUAD. Inhibition of ERCC8 demonstrated antitumor effects on multiple scales, including proliferation, apoptosis, and metastasis abilities *in vitro.* Given the limited exploration of ERCC8’s biological function, further investigation is needed to unveil the underlying mechanisms driving its impact on LUAD. Additionally, evidence from animal experiments is also necessary for the evaluation of the clinical value of ERCC8 as a newly identified therapeutic target.

Given the critical role of ERCC proteins in cancer cell chemoresistance, inhibiting their function or disrupting their interactions with partner proteins represents a potential strategy for anti-cancer therapy. Indeed, small-molecule inhibitors that block the interaction between ERCC1 and XPF—two key factors in the nucleotide excision repair (NER) pathway—can enhance the cytotoxic effects of cisplatin and mitomycin C in lung cancer and colorectal carcinoma cells (42, 43). Similarly, identifying inhibitors for other ERCC proteins, such as ERCC8, may offer therapeutic benefits for LUAD patients.

In summary, our study, through extensive data mining and bioinformatic analyses, has established the comprehensive functional landscape of the effect of the ERCC family on LUAD development. Furthermore, we have validated the oncologic function of ERCC8 in LUAD and implied its potential as a novel target for this highly heterogeneous tumor with chemotherapy resistance.

## Data Availability

The original contributions presented in the study are included in the article/**Supplementary Material**. Further inquiries can be directed to the corresponding author.
