# Supplementary material for: Characterization of the function and clinical value of ERCC family genes in lung adenocarcinoma
Source: Front Oncol. 2024 Nov 8;14:1476100. doi: 10.3389/fonc.2024.1476100 (PMC11581973; doi:10.3389/fonc.2024.1476100)
Supplement: Supplementary file 1 [file DataSheet1.docx]

**
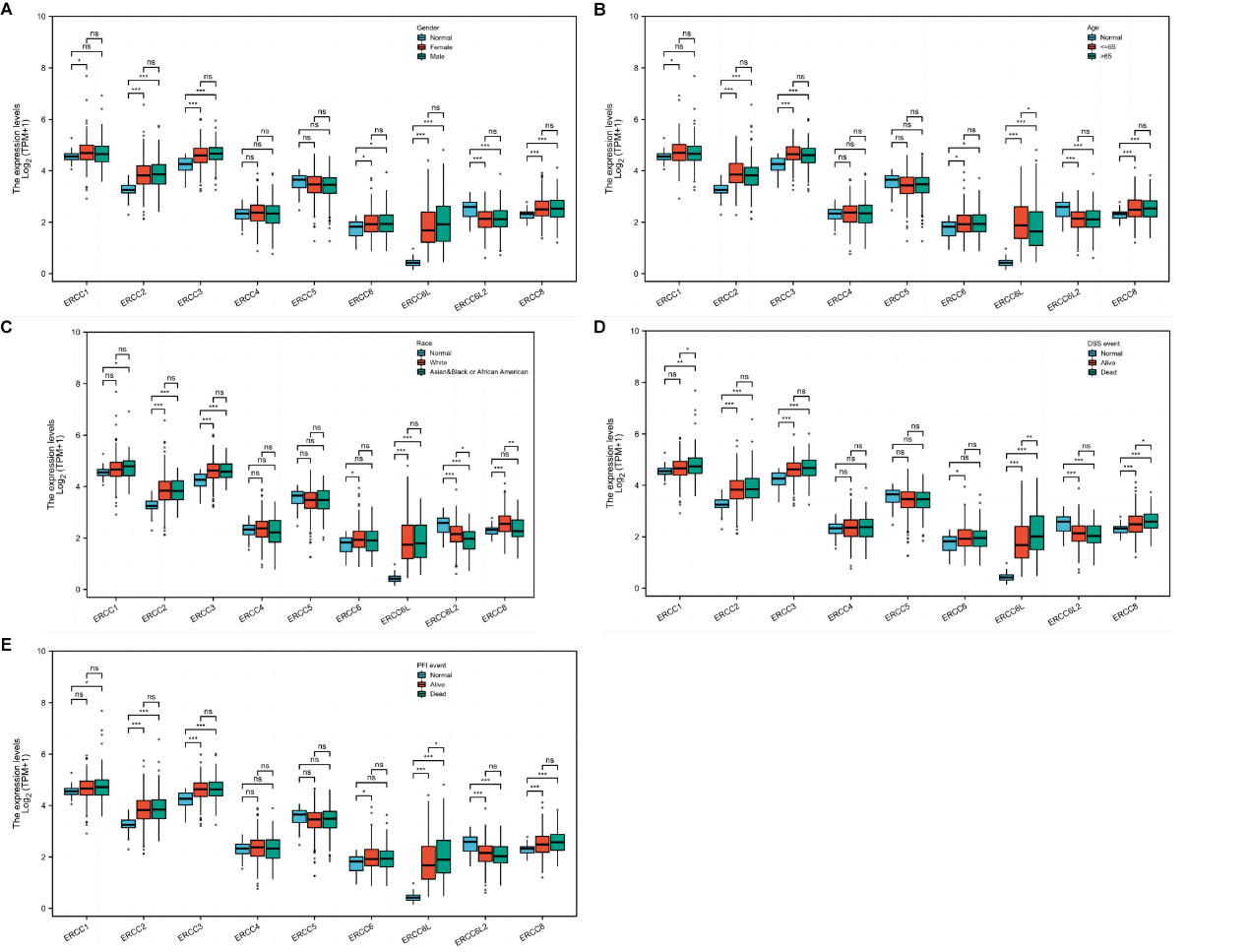
**

**Figure S1. Expression levels of ERCC genes in LUAD.** A-E. Comparison of ERCC gene expression level in LUAD of different groups classified according to gender (A), age (B), race (C), DSS (D), or PFI (E).


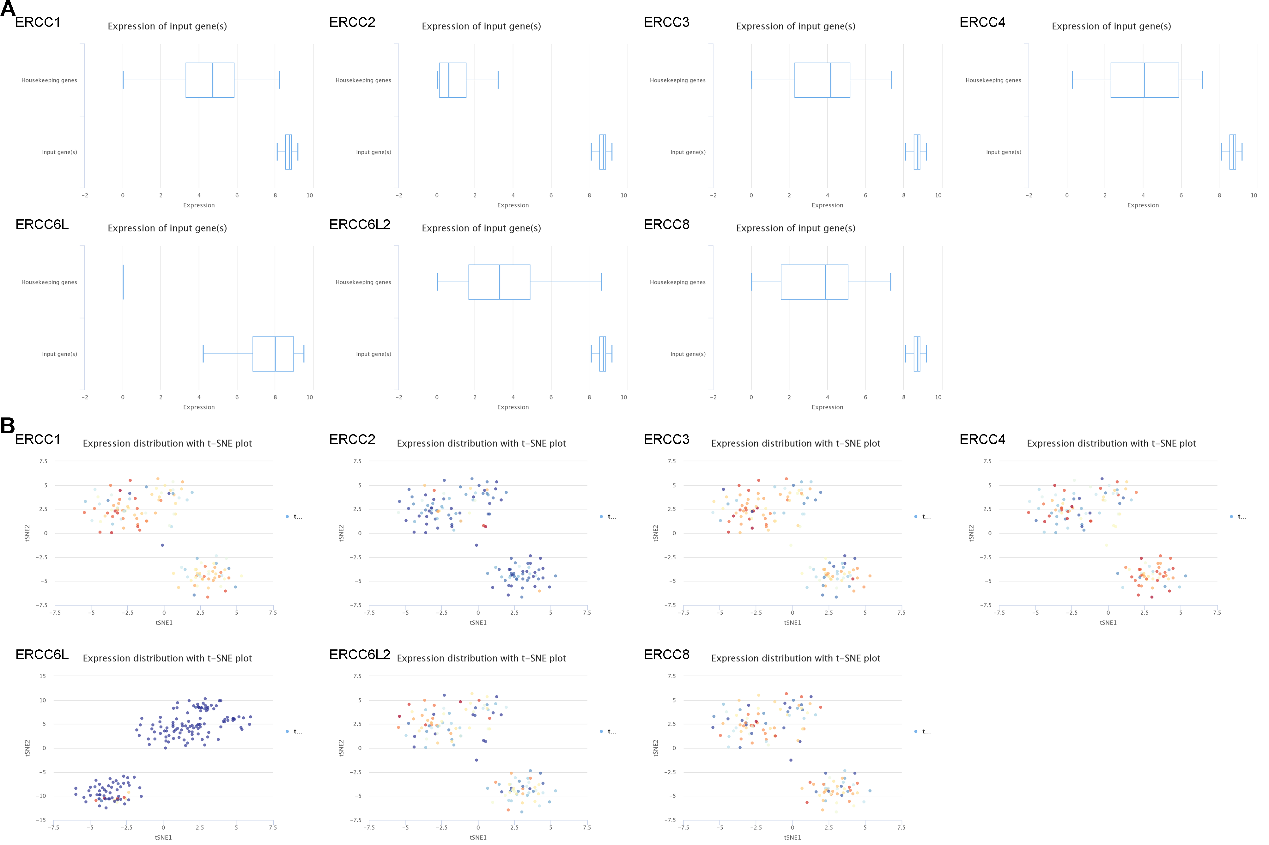


**Figure S2. Single-cell scale analysis of ERCC family in LUAD.** A. Box diagrams indicating the expression of ERCC family genes in LUAD in single-cell sequencing datasets. B. t-SNE representation of cell clusters in LUAD after multidimensional scaling and dimensionality reduction. Each point represents a single cell, and the color of the point represents the expression level of the gene indicated beside the figure, which displays the distribution of heterogeneous LUAD cells together with ERCC gene expression.


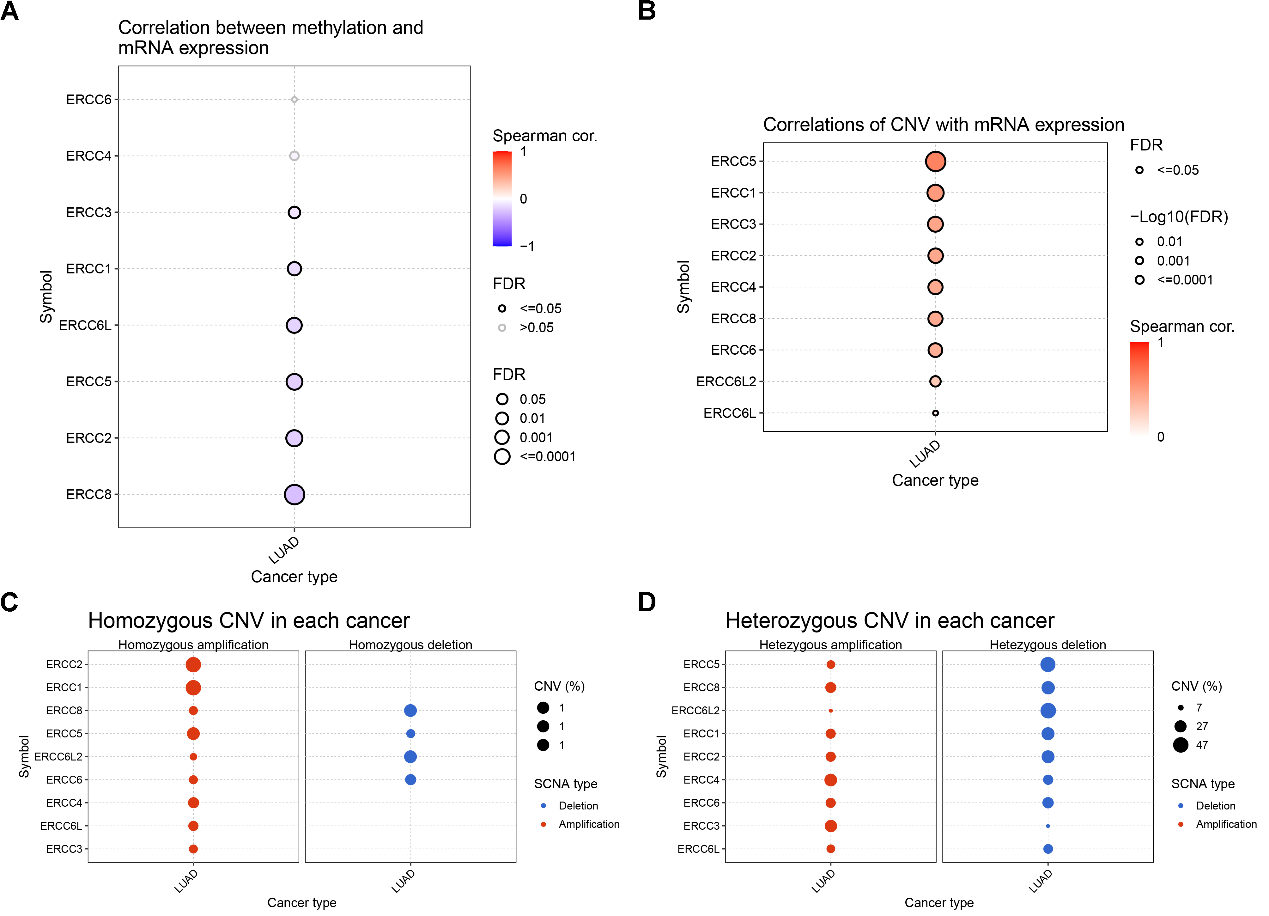


**Figure S3. Correlation of methylation and CNV of ERCC genes with their expression in LUAD.** A. Summary of correlation and FDR between methylation and gene expression from Figure 5. B. Summary of correlation and FDR between CNV and gene expression from Figure 6. C and D. Distribution of homozygous (C) and heterozygous (D) CNV among ERCC family genes in LUAD samples. The size of the circle indicates the proportion of CNV occurrence in all samples analyzed.


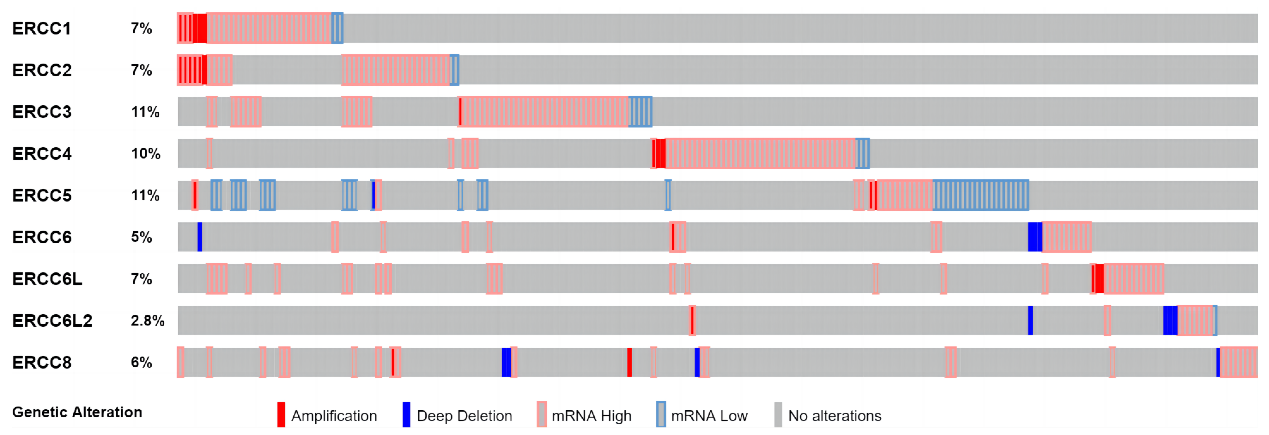


**Figure S4. CNV and expression level distribution among samples.** Distribution of gene amplification, deep deletion, mRNA increase, and decrease of ERCC family genes in LUAD samples. Each block represents one sample.

**
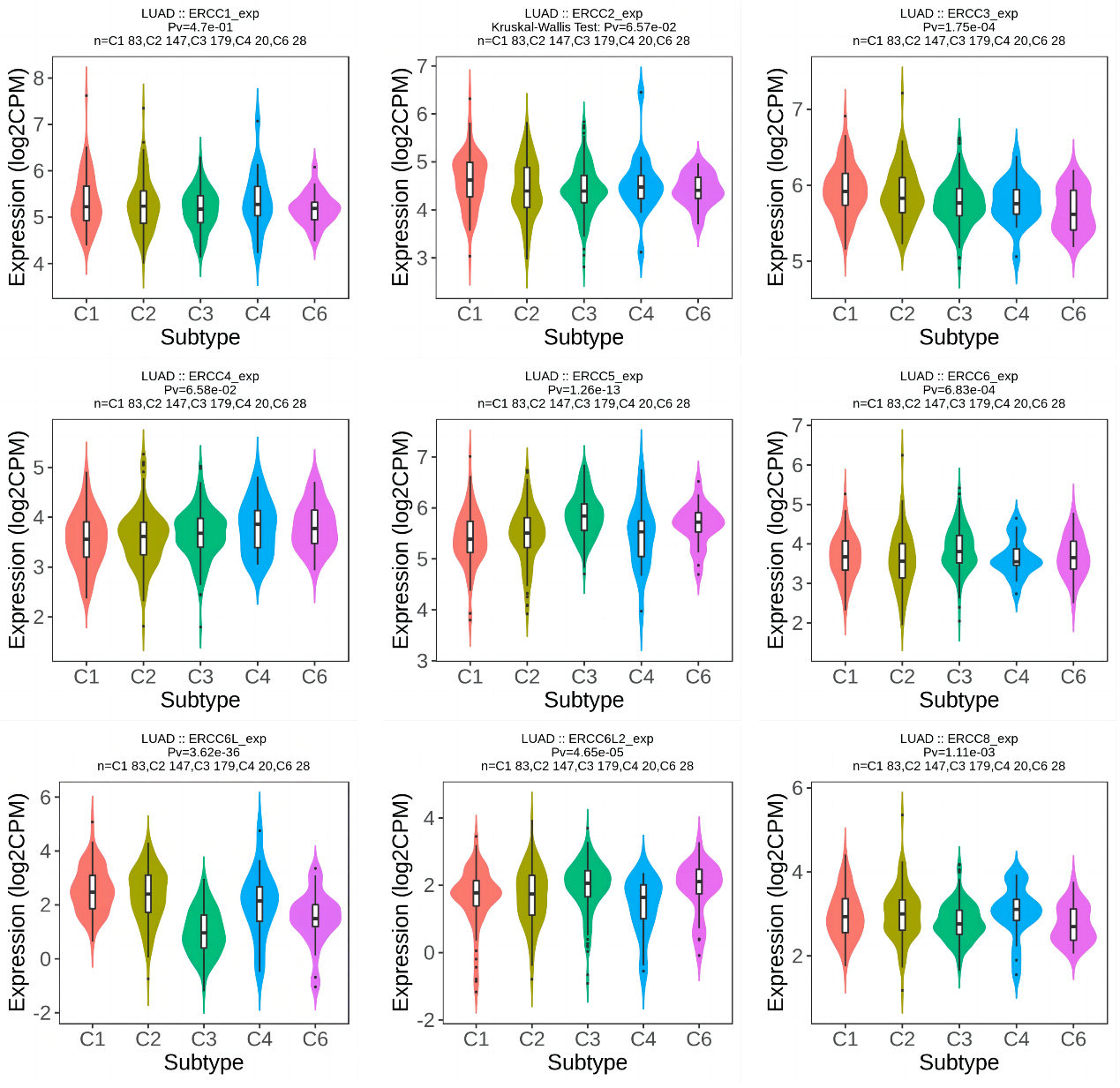
**

**Figure S5. Expression levels of ERCC genes in different subtypes of LUAD.** Violin plots displaying the expression distribution of ERCC genes in each tumor subtype in LUAD analyzed using TISIDB. C1: wound healing; C2: IFN-γ dominant; C3: inflammatory; C4: lymphocyte depleted; C6: TGF-β dominant.


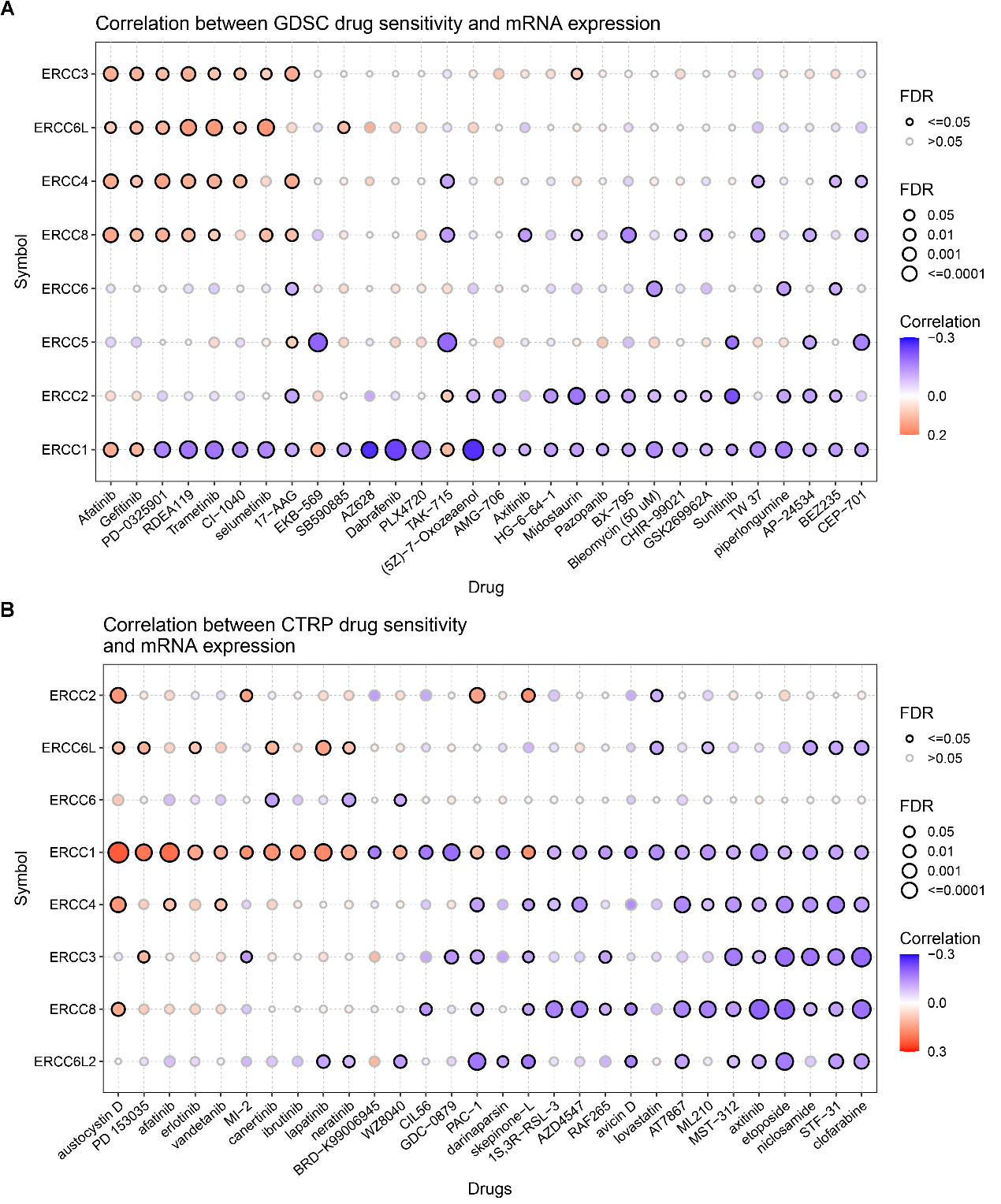


**Figure S6. Correlation between ERCC gene expression and drug sensitivity in LUAD.** A and B. Plots indicating the correlation between ERCC gene expression with sensitivity of drugs from GDSC (A) and CTRP (B) catalogs in LUAD.


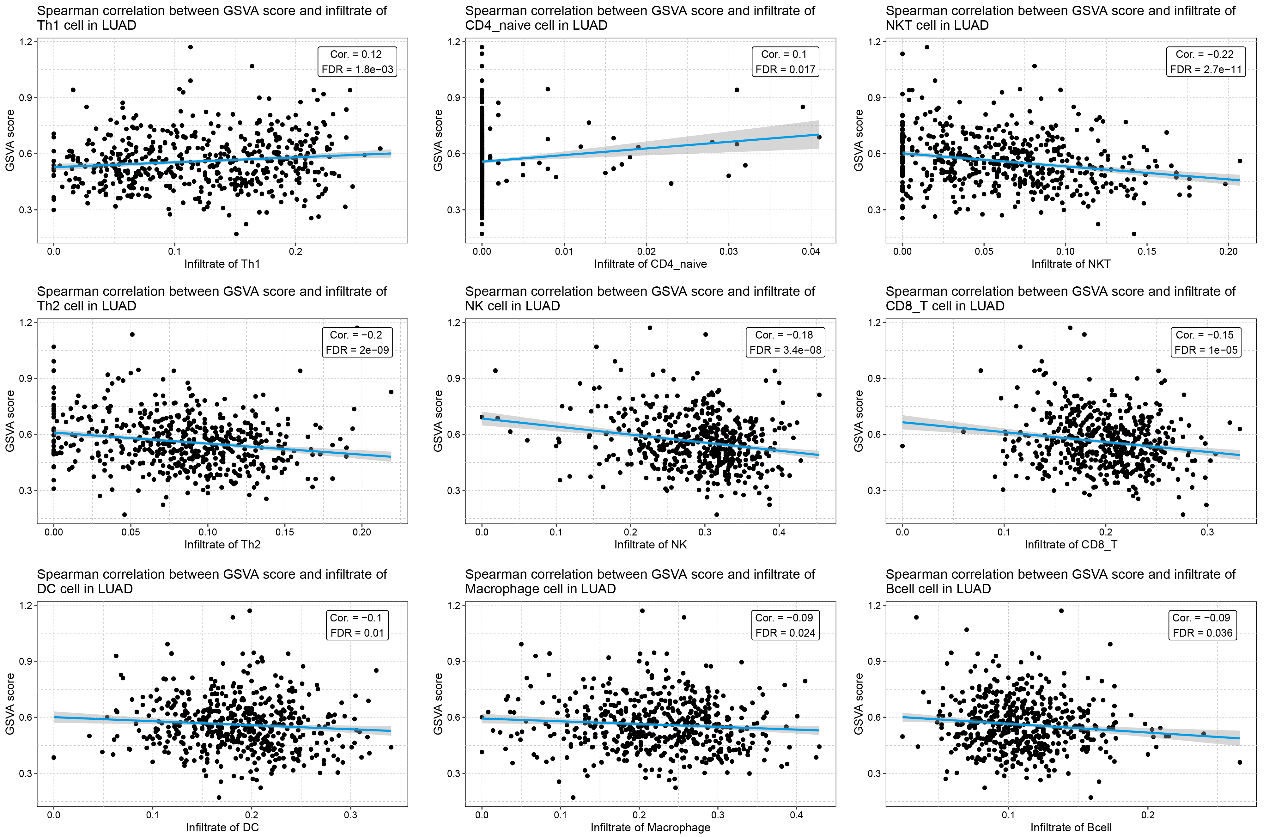


**Figure S7. Association between GSVA score and TILs in LUAD.** Spearman correlation displaying the other cell types with significant correlations that were not shown in Figure 11. *: Cor > 0.1 or < -0.1; #: FDR < 0.05.
